# Brain Bases of Working Memory for Time Intervals in Rhythmic Sequences

**DOI:** 10.3389/fnins.2016.00239

**Published:** 2016-06-01

**Authors:** Sundeep Teki, Timothy D. Griffiths

**Affiliations:** ^1^Wellcome Trust Centre for Neuroimaging, University College LondonLondon, UK; ^2^Institute of Neuroscience, Newcastle UniversityNewcastle upon Tyne, UK

**Keywords:** interval timing, time perception, working memory, rhythm, fMRI

## Abstract

Perception of auditory time intervals is critical for accurate comprehension of natural sounds like speech and music. However, the neural substrates and mechanisms underlying the representation of time intervals in working memory are poorly understood. In this study, we investigate the brain bases of working memory for time intervals in rhythmic sequences using functional magnetic resonance imaging. We used a novel behavioral paradigm to investigate time-interval representation in working memory as a function of the temporal jitter and memory load of the sequences containing those time intervals. Human participants were presented with a sequence of intervals and required to reproduce the duration of a particular probed interval. We found that perceptual timing areas including the cerebellum and the striatum were more or less active as a function of increasing and decreasing jitter of the intervals held in working memory respectively whilst the activity of the inferior parietal cortex is modulated as a function of memory load. Additionally, we also analyzed structural correlations between gray and white matter density and behavior and found significant correlations in the cerebellum and the striatum, mirroring the functional results. Our data demonstrate neural substrates of working memory for time intervals and suggest that the cerebellum and the striatum represent core areas for representing temporal information in working memory.

## Introduction

Everyday we are required to assess sequences of variable time intervals that occur in sounds like speech, music and environmental sounds, a process that requires us to hold multiple time intervals in memory. This work examines the neural bases for holding time intervals in working memory and the effect of changing the amount of information in these sequences determined by the temporal variability and number of intervals.

The nature of working memory in general is under debate (Ma et al., 2014). Classical visual models assume a limited working memory capacity (Miller, 1956; Cowan, 2001) where information is stored in a fixed number of discrete slots (Luck and Vogel, 1997). However, recent visual and auditory studies support a resource allocation model based on a limited working memory resource that is dynamically distributed between multiple items in natural scenes, without a slot limit (Bays and Husain, 2008; Gorgoraptis et al., 2011; van den Berg et al., 2012; Kumar et al., 2013; Ma et al., 2014; Teki and Griffiths, 2014; Joseph et al., 2015a,b, 2016). Neither of these models, however, has considered the question of how time intervals are held in working memory.

We designed a novel paradigm to assess working memory for sequences of intervals that systematically changed the information held in working memory and examined working-memory fidelity (Teki and Griffiths, 2014). Listeners were presented with sequences that consisted of two types of sequences: (1) sequences with a fixed number of intervals with different levels of temporal regularity, and, (2) sequences with a varying number of intervals with a fixed temporal regularity. The task did not involve a binary response (e.g., shorter/longer or same/different judgment) about the probed interval change as in previous studies, but instead required the participant to reproduce the duration of a single interval that was probed after the sequence. This allowed us to examine the effects of the variability and number of intervals in the sequence on the precision (reciprocal of standard deviation) for probed interval reproduction (Teki and Griffiths, 2014). The results are consistent with a working memory model based on a fixed resource for storing time intervals so that a greater numbers of intervals can be stored at the expense of fidelity (Bays and Husain, 2008). The present study sought to address the neural bases for the core working memory resource, determined by both temporal variability and number of intervals.

Previous work on memory for time was either based on retention of a single interval into memory for subsequent comparison or involved multiple presentations of a standard interval that formed an isochronous sequence (Keele et al., 1989; Ivry and Hazeltine, 1995; Merchant et al., 2008). Other studies used induction sequences to study the effect of rate of presentation of those sequences (Barnes and Jones, 2000) or the temporal structure of the sequence (McAuley and Jones, 2003; Teki et al., 2011) on judgments of the duration of subsequent intervals. However, as these studies involved repetition of standard intervals, the effective memory load was limited to the interval used as the basis for the induction sequence.

Previous imaging work has shown that the putamen and caudate nucleus encode the duration of single time intervals (Rao et al., 2001; Coull et al., 2008) while recent work suggests that areas for the analysis of single intervals alter with the sequence context (Merchant et al., 2013). Timing in regular sequences relies more on a striato-thalamo-cortical network whilst timing in irregular sequences depends more on the cerebellum (Grahn and Rowe, 2009; Teki et al., 2011, 2012; Kung et al., 2013; Allman et al., 2014). The present study addresses brain bases for storing time intervals in memory as is required for natural acoustic stimuli, for which we hypothesized a striatal and cerebellar substrate.

Another motivation of the study was to examine contextual factors: the effect of task context on stimuli with the same variability and number of intervals. Previous work was mostly based on single intervals and thus could not address this crucial question. Recent reviews emphasize task-dependent activation of brain areas associated with temporal processing (Wiener et al., 2010a; Merchant et al., 2013) but there are no data suggesting that the activity of brain areas underlying memory for time intervals may also be modulated by task context.

We used functional magnetic resonance imaging to uncover the neural substrates that represent sequences of intervals in working memory. Our results highlight activity in core perceptual timing areas including the cerebellum and the striatum that varies with the amount of information in a sequence, determined by temporal regularity and number of intervals. Holding and manipulating the same interval in working memory depended on the context, the number of intervals in the sequence, in the caudate nucleus and the inferior parietal cortex. Our data support the flexible representation of time intervals in working memory where the cerebellum and caudate provide the core resource.

## Materials and methods

### Participants

Nineteen listeners (12 females; mean age: 27.4 ± 2.3 years) with normal hearing and no history of audiological or neurological disorders provided informed written consent and participated in the experiment. A female listener was excluded from the analysis due to excessive movement in the scanner. Two listeners could not complete the number-of-interval blocks. Thus, 18 listeners provided datasets for the jitter condition whilst 16 listeners' datasets were analyzed for the number-of-intervals condition. All but four listeners had musical experience but none of them were currently practicing music. Experimental procedures were approved by the research ethics committee of University College London.

### Stimuli

**Figure 1:**
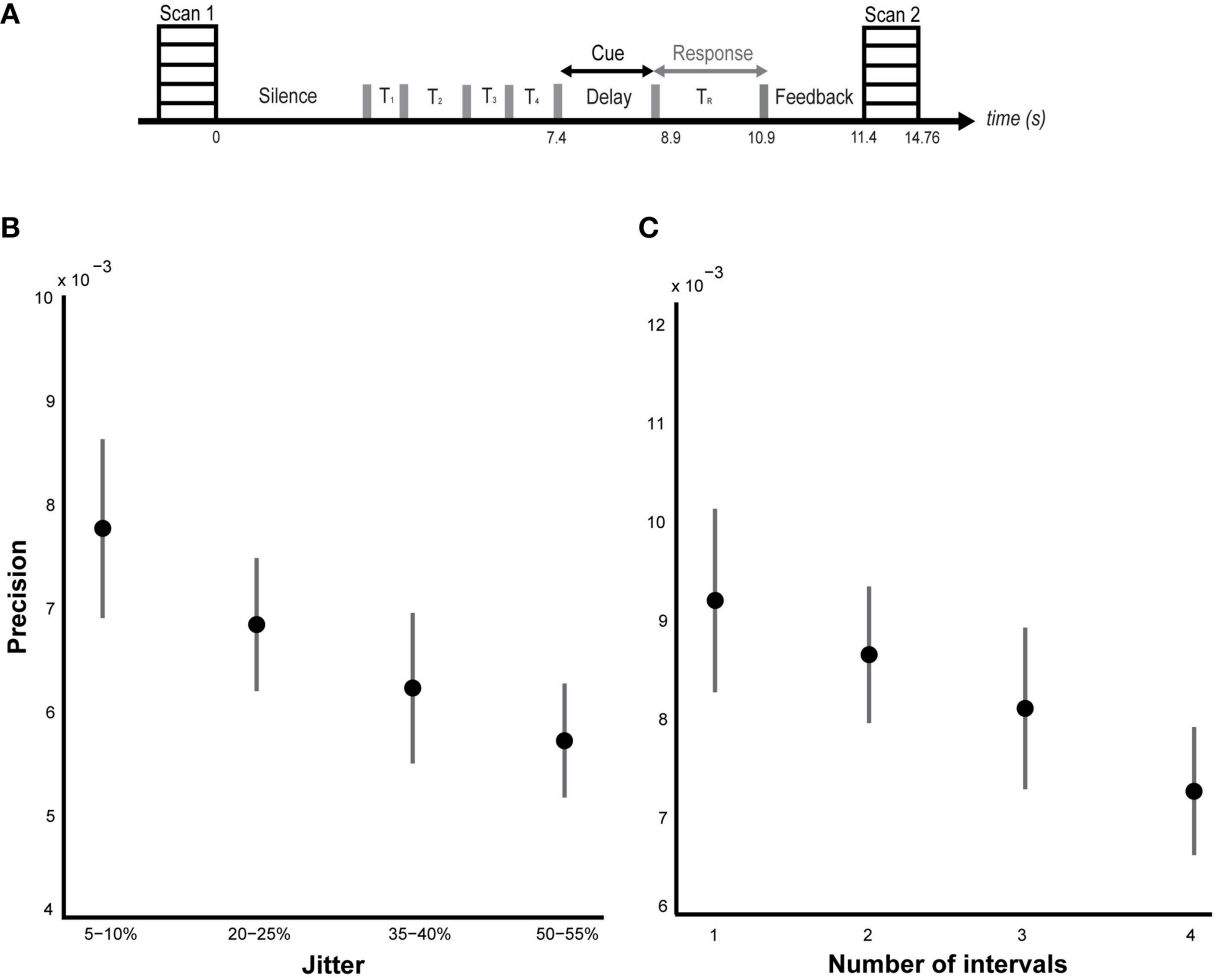
**Behavioral results. (A)** Task and sparse imaging paradigm. Listeners are presented with a sequence of time intervals (4 intervals in the jitter condition and 1–4 intervals in the number-of-intervals condition) separated by clicks (indicated by the gray bars). At the end of the sequence, a probe is presented during the delay period (2 s) indicating the interval to be reproduced. Another click is played after the delay indicating the start of the reproduction interval which listeners are required to terminate at a point in time that corresponds to their memory of the probed interval. Feedback, equal to the difference between the reproduced interval and the actual duration of the probed interval is presented for 500 ms at the end of each trial. The task structure and timing is shown between two successive volumes in the sparse imaging design. **(B)** Performance on jitter blocks. Listeners' performance (*n* = 18) was calculated as the precision of the timing error distribution. The mean precision (± SEM) is plotted as a function of temporal regularity, varying from 5–10% jitter to 50–55% jitter as indicated on the x-axis. A significant effect of jitter (*p* = 0.02) on precision was observed (see results). **(C)** Performance on number of intervals blocks. Listeners' (*n* = 16) mean precision (± SEM) is plotted against the number of intervals on the abscissa. No significant effect of memory load (*p* = 0.36) was found (but see results).

The stimulus (Figure 1) consists of a sequence of clicks of 0.5 ms duration and identical loudness. The inter-onset interval (IOI) was selected from a normal distribution that ranged from 500 to 600 ms. For the *Jitter* blocks, the stimulus comprised four time intervals. By jitter, we refer to variability in the length of a time interval around a mean value of inter-onset interval. For instance, introducing a 10% jitter for a 100 ms interval would yield an interval whose duration may vary from 90 to 110 ms. Four different levels of temporal jitter were incorporated: (i) 5–10%, (ii) 20–25%, (iii) 35–40%, and, (iv) 50–55%. Higher jitter values enhance the difference in duration between the various intervals and make each interval more unique, thereby increasing the memory load. The exact jitter values were randomly drawn from a normal distribution centered on the mean of each of the four ranges of jitter. Each sequence block was jittered by only one of the above ranges of jitter.

The stimuli for the *Number-of-intervals* blocks consisted of sequences with different number of time intervals, from 1 to 4, and a fixed jitter of 20–25%. The stimulus for the reaction time task consisted of a single click only.

Stimuli were created digitally using MATLAB 2012 (MathWorks Inc.) at a sampling rate of 44.1 kHz and resolution of 16 bits. Sounds were delivered diotically through MRI compatible insert earphones (Sensimetrics Corp.) and presented at a comfortable listening level between 80 and 90 db SPL that was adjusted by each listener. The stimulus presentation was controlled using Cogent (http://www.vislab.ucl.ac.uk/cogent.php).

### Timing task

The task was designed to assess listeners' memory for time intervals embedded in sequences in which the temporal jitter and number of intervals were varied parametrically (Teki and Griffiths, 2014). Listeners were instructed to attend to the sequence of clicks and reproduce the duration of interval that was probed after the sequence (via text displayed on the screen—e.g., “Match time interval: 1”). The probed interval number was displayed during the entire delay after the sequence period lasting 1.5 s. A click was played after the delay period and indicated the start of the interval to be reproduced. The listeners' task was to press a button at a point in time (after this click) that corresponded to their memory of the duration of the probed interval. Responses made within a window of 2 s were considered valid responses while responses longer than 2 s were treated as “missed” responses. Feedback, equal to the difference between the duration of the probed interval and the listeners' response (adjusted for reaction times) was presented for 500 ms after each trial (e.g., “Shorter by 53.2 ms” or “Longer by 107.4 ms”).

### Control task

A control task was used prior to each timing block to calculate listeners' response times to a single click. The reaction times were used to regress out variance due to the motor response from the time matching responses in the experimental blocks.

### Procedure

Listeners received instructions about the task and practiced a reaction time block of 15 trials and a jitter block of 24 trials. Training was repeated until performance improved, as assessed by precision values. However, participants did not receive any explicit information or training for the number-of-interval blocks.

In order to investigate context-sensitive responses, listeners only received training on the jitter blocks and not the (later) number-of-interval blocks. It was important to not counterbalance the order of the jitter and number-of-interval blocks to ensure that listeners held only one task context in mind during the jitter block and then switched to a different task context provided by the number-of-interval blocks. Listeners received brief training on the number-of-intervals condition in the scanner after the jitter blocks were completed. This enabled us to compare brain activations for trials that were identical in structure (32 trials with 20–25% jitter and 4 intervals in a sequence) across the two task conditions.

The task of the listeners was to reproduce the duration of the cued interval from memory by pressing a button on a keypad. Responses were always made with the index finger and the use of right and left hand was counterbalanced across participants. Prior to each timing block, listeners completed a reaction time block comprising 30 trials where they pressed a button in response to a single click. Listeners were instructed to respond at a comfortable rate and maintain the same pace for both the reaction time and timing trials throughout the experiment.

The imaging experiment lasted ~1 h and consisted of two jitter blocks (varying jitter and fixed number of intervals) followed by two number-of-intervals blocks (varying number of intervals and fixed jitter) where each block consisted of 64 trials. Field maps were acquired after the first two blocks and listeners were instructed about the change in stimulus structure and received limited training on the number-of-intervals condition whilst in the scanner. Each block lasted ~15 min and short breaks were allowed between successive blocks. Listeners were instructed to keep their eyes open as the probed interval was indicated visually on the screen. At the end of each block, listeners received feedback specifying the number of trials on which their timing error was less than 100 ms, between 100 and 200 ms, or greater than 200 ms. A structural scan was acquired at the end of the functional imaging experiment for each participant.

### Behavioral analysis

The median of the reaction times for the final 24/30 trials was computed for each reaction time block. For the timing blocks, the error response was calculated as the difference between the time matching response and the actual duration of the cued interval. The median reaction time from the preceding control (reaction time) block was subtracted from this value. This allowed us to obtain a cleaner measure of the time matching response that was not confounded with the time taken for button press (see Teki and Griffiths, 2014). This analysis was repeated for each timing block.

The absolute value of the error responses was used to calculate precision, by computing the inverse of the standard deviation of the error responses. Precision was measured as a function of jitter and as a function of number of intervals for the corresponding blocks. Precision was used as the primary measure of interest as it captures the true variability in memory performance. This is useful to interpret variability in performance with increasing number of items and examine whether performance is fixed up to a certain number of slots (according to slot models) or scales flexibly according to the total amount of information to be remembered (according to shared resource models). The slot model would predict that the precision would be at ceiling for a set number of items such as four (see Cowan, 2001) until capacity is exceeded and would drop to floor for a set size that exceeds the working memory capacity. The shared resource model, however, predicts that precision is highest for a set size of one and decays as a function of the number of items to be remembered (Ma et al., 2014). Crucially, the precision for higher memory loads greater than four is predicted to be higher than that obtained by chance. Absolute error or accuracy measures do not capture such variability and are thus not ideal for comparing the two models.

### Image acquisition

Gradient weighted whole-brain echo planar images were acquired using a 3T Siemens Allegra system using a sparse imaging design: time to repeat (TR) of 14.76 s; time to echo: 30 ms (TE); time for volume acquisition (TA): 3.36 s (70 ms to acquire one slice × 48 slices); matrix size: 64 × 72; slice thickness: 2 mm with 1 mm gap between slices; and, in-plane resolution: 3.0 × 3.0 mm^2^. The slices were tilted by −7° (transverse > coronal) to obtain full coverage of the cerebellum. This orientation was used successfully to uncover perceptual timing responses in the inferior olive and the cerebellum in our previous fMRI timing study (Teki et al., 2011). Field maps were acquired to compensate for geometric distortions due to magnetic field inhomogeneity (Hutton et al., 2002) using a double-echo gradient echo field map sequence (TE_1_ = 10.00 ms and TE_2_ = 12.46 ms). A T1-weighted structural scan was acquired after the functional scans (Deichmann et al., 2004).

A sparse sampling design (Figure 1) was used to obtain clean auditory activations unaffected by the scanner noise (Belin et al., 1999). The total duration of the stimulus ranged from 0.5 to 2.6 s depending on the number of intervals (1–4) in the sequence. A variable silence period preceded the onset of the stimulus such that the combined duration of silence and stimulus was fixed at 7.4 s. A delay period of 1.5 s, response window of 2 s and a feedback period of 0.5 s, in that order, completed each trial with a fixed duration of 11.4 s. The latency between trial offset and scanner onset was fixed at 4 s so that the acquisition of each scan was time-locked to the onset of the delay period. This latency of 4 s was based on our previous study where we used a similar sparse imaging protocol to isolate timing responses in the cerebellum and the striatum (Teki et al., 2011). The fixed latency helped ensure that the peak of the BOLD signal captured brain activity corresponding to the manipulation and retrieval of the cued interval from memory rather than earlier stimulus-evoked or subsequent motor activity, with minimal overlap in their hemodynamic response functions (HRFs). Given the poor temporal resolution of fMRI, one cannot be completely confident about the extent to which the scan acquired at the end of each trial was contaminated by effects not related to memory processes during the delay period. However, the manipulation of keeping a fixed latency from the onset of the delay period to the onset of the acquisition of the scan is motivated by the characteristic latency of BOLD responses to sounds in sparse imaging protocols (~4 s, Belin et al., 1999; Hall et al., 1999) and is a reliable method to obtain pseudo time-locked responses using sparse fMRI (Teki et al., 2011; Talavage et al., 2014).

### Image analysis

The analysis of brain imaging data was performed using SPM12 (Wellcome Trust Centre for Neuroimaging, Ashburner, 2012). Each block comprised of 66 volume acquisitions out of which the first two volumes were rejected to control for saturation effects. The remaining 64 volumes were realigned to the first volume and unwarped using field map parameters. The structural image was segmented to obtain a bias-corrected structural image that has more uniform intensities within six different tissue classes including gray matter (GM) and white matter (WM). The resulting image was co-registered with the mean functional image obtained after realignment. DARTEL was used to create a series of templates using the GM and WM images (Ashburner, 2007). The final template from this step was affine registered with tissue probability maps (available in SPM12) to obtain spatially normalized images in MNI space (Friston et al., 1995a). The normalized images were smoothed with an isotropic Gaussian kernel of 5 mm full-width at half-maximum (FWHM).

Statistical analysis of the images was performed using general linear model (Friston et al., 1995b). Data from the jitter and number-of-interval blocks were analyzed separately using a parametric contrast to examine brain activity that increased as a function of jitter and number-of-intervals respectively. All trials were convolved with an HRF boxcar function and missed trials were modeled as conditions of no interest (separately for each condition) to remove unwanted variance. The data were not high-pass filtered as a sparse design ensures minimal low-frequency variations in the BOLD signal.

A whole-brain random-effects model was used to account for within-subject variance (Penny and Holmes, 2004). Each subject's first-level contrast images were subjected to second-level *t*-tests for the primary contrasts of interest: “parametric effect of jitter” and “parametric effect of number of intervals.” To examine context-dependent memory encoding for trials that were identical in the two conditions, a separate design based on difference in activations between the jitter versus number-of-interval blocks (and vice-versa) was used. Functional data were visualized on the group-averaged T1-weighted structural scan and activations specific to the cerebellum were overlaid on the high-resolution, spatially unbiased infra-tentorial template (SUIT) atlas of the human cerebellum (Diedrichsen, 2006; Diedrichsen et al., 2009).

Structural brain images were analyzed using voxel-based morphometry (VBM; Ashburner and Friston, 2000). The segmented GM and WM images were imported into DARTEL and a series of template images were created by iteratively matching images to align them with the average-shaped template. The final template obtained in this procedure was normalized to MNI space through an affine registration of the template with tissue probability maps. The resultant images were smoothed with an isotropic Gaussian kernel of 8 mm FWHM. The smoothed images for each individual were entered into a second-level ANOVA to examine brain areas in which GM and WM volume varied as a function of jitter and number of intervals respectively.

## Results

### Behavioral results

Participants' performance in the scanner was measured by calculating precision, the inverse of the variance of the timing error distribution for both blocks. Precision provides a continuous measure of memory performance and has been used previously in studies of working memory based on the shared resource model (Bays and Husain, 2008; Bays et al., 2009; Kumar et al., 2013; Ma et al., 2014; Teki and Griffiths, 2014; Joseph et al., 2015a,b, 2016).

ANOVA revealed a main effect of jitter (*p* = 0.02, *F* = 3.40, η^2^ = 0.14) but a non-significant effect of number of intervals [*p* = 0.36, *F*_(3, 63)_ = 1.10, η^2^ = 0.05] as shown in Figures 1B,C respectively. *Post-hoc* analysis revealed a significant difference between the precision for the least and most irregular conditions in the jitter experiment: *p* = 0.048, *t* = 2.05; and a marginal but not significant difference between the precision for the trials with lowest and highest number of intervals: *p* = 0.10, *t* = 1.69. Secondary analysis of precision as a function of serial position did not reveal a significant effect for either condition: *p* = 0.10, *F* = 2.14, η^2^ = 0.09 (jitter block), *p* = 0.38, *F* = 1.05, η^2^ = 0.05 (number of intervals block).

Although a significant effect of number of intervals was not observed during performance in the scanner, our previous psychophysical work did demonstrate a significant effect: [*p* = 0.01, *F*_(3, 28)_ = 4.27, η^2^ = 0.31, *n* = 8; Teki and Griffiths, 2014]. The absence of a behavioral effect in the scanner could be due to a number of reasons: (i) listeners did not receive explicit and adequate training about the number-of-intervals blocks before the experiment, (ii) the number-of-interval blocks were always run after the jitter blocks and could be associated with increased fatigue, (iii) reduced number of trials in the scanner: 2 blocks of 64 trials compared to 4–5 blocks of 96 trials in the psychophysics study, (iv) limited response time and a noisier task environment in the scanner. Further investigation of individual behavioral scores in the number-of-interval blocks revealed the opposite trend in 4 subjects who showed no significant effect: [*F*_(3, 15)_ = 0.66, *p* = 0.59, η^2^ = 0.14]. A similar ANOVA on the scores of the remaining 12/16 subjects revealed a significant effect of number of intervals: [*F*_(3, 47)_ = 2.84, *p* = 0.04, η^2^ = 0.16].

### Functional imaging results

We analyzed BOLD responses to examine brain areas that: (i) encode memory for time as a function of increasing and decreasing jitter, (ii) are activated as a function of increasing and decreasing numbers of intervals, and (iii) the effect of task context in modulating brain activity in response to identical trials across the two conditions.

*A priori*, we predicted that both cerebellum and striatum would show increased activity as a function of increasing as well as decreasing jitter, but with opposite effects such that cerebellum would be more strongly activated for encoding temporal memory in irregular sequences and the striatum would show elevated activity for regular sequences (Grahn and Brett, 2007; Teki et al., 2011, 2012; Grahn, 2012; Merchant et al., 2013). Secondly, based on previous fMRI work on temporal memory encoding (Rao et al., 2001; Coull et al., 2008), we hypothesized that the striatum would be involved in encoding memory for time as a function of increasing numbers of intervals. Thirdly, we expected that task context would modulate brain activity such that areas that represent the structure of sequences of intervals would show differential responses for trials that were identical in structure during the jitter and number-of-intervals conditions.

#### Effect of jitter

**Figure 2:**
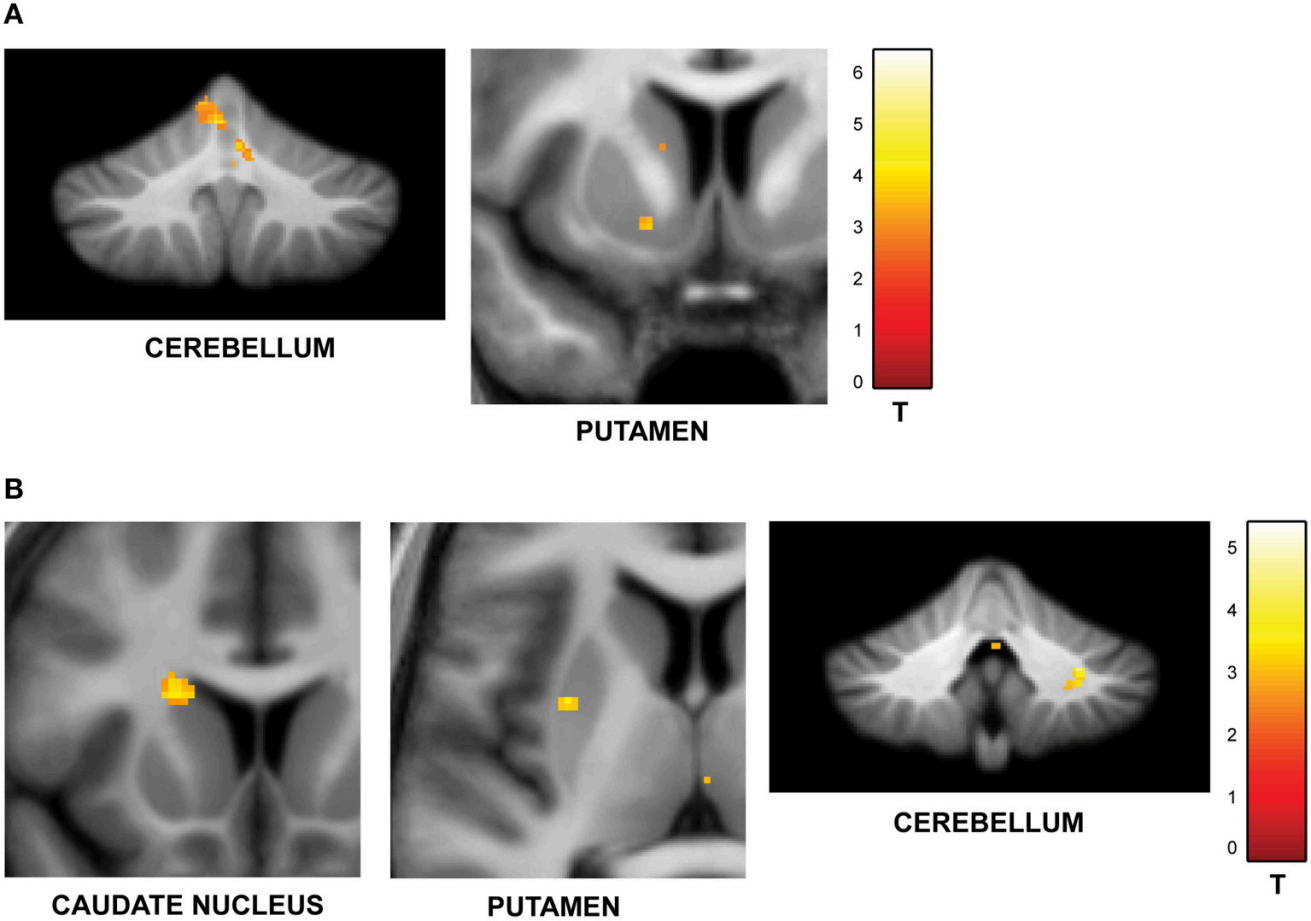
**Functional imaging results: effect of jitter. (A)** Brain areas that encode temporal memory in the context of irregular sequences. BOLD activations are shown for the vermis and cerebellum (overlaid on the SUIT template of the human cerebellum, Diedrichsen, 2006, Diedrichsen et al., 2009); left putamen and parahippocampal gyrus (overlaid on a coronal section of the average normalized structural scan and zoomed to 80 × 80 mm) at a threshold of *p* < 0.001 (uncorrected, for each figure). Other activations in the precuneus, MTG, and pallidum are listed in Table 1A. The strength of activations (*t*-value) is graded according to the adjacent color scheme on the right (for each figure). **(B)** Brain areas that encode temporal memory in the context of regular sequences. BOLD activations in the striatum including the caudate and putamen as well as the cerebellum are shown. Other activations in the thalamus, temporal pole, and frontal cortex are listed in Table 1B. The significant clusters are displayed according to the same scheme as in Figure 1A.

To answer the first question, data from the blocks with different levels of jitter were analyzed. A parametric contrast was used to examine areas that showed an increase in response as a function of increasing jitter. Results revealed significant clusters in the left cerebellum (lobules I-IV, V) including the vermis as shown in Figure 2A. The striatum was also significantly modulated, with clusters in the putamen and pallidum. Other brain areas whose activity was significantly modulated by increasing levels of jitter included the precuneus, the parahippocampal gyrus and the middle temporal gyrus (see Table 1A).

**Table 1A:** **Brain areas whose activity increased as a function of jitter**.

**Brain area**	**Hemisphere**	***x***	***y***	***z***	***t*-value**
Vermis	Left	−5	−42	−36	4.86
	Right	5	−51	17	4.09
Cerebellum lobule I-IV, V	Left	−3	−56	−12	4.65
Cuneus/Precuneus	Right	9	−74	20	4.60
	Left	−9	−57	42	3.94
Parahippocampal Gyrus	Left	−20	−24	−26	3.85
Middle Temporal Gyrus	Right	45	−33	−6	3.81
Putamen	Left	−15	8	−9	3.69
Pallidum	Left	−18	−1	12	3.67

*Local maxima are shown at p ≤ 0.001 (uncorrected)*.

Examination of parametric responses in the opposite direction (as a function of decreasing jitter) showed maximum activation in the striatum including the caudate and putamen (Figure 2B). We also observed activity in the cerebellum (right posterior lobe); however, the strength of the activation in the cerebellum was weaker than the striatal response (see Table 1B). The frontal cortex, temporal pole and thalamus also showed significant activations with decreasing levels of jitter.

**Table 1B:** **Brain areas whose activity decreased as a function of jitter**.

**Brain area**	**Hemisphere**	***x***	***y***	***z***	***t*-value**
Caudate nucleus	Right	−20	18	18	5.36
	Left	24	5	26	5.26
Putamen	Right	33	0	−5	4.37
	Left	−29	−2	8	3.50
Cerebellum lobule VIII	Right	29	−48	−36	4.49
Frontal cortex	Right	26	−20	32	5.37
Temporal pole	Right	41	17	−24	4.03
Thalamus	Right	17	−12	3	3.67

*Local maxima are shown at p ≤ 0.001 (uncorrected)*.

#### Effect of number of trials

**Figure 3:**
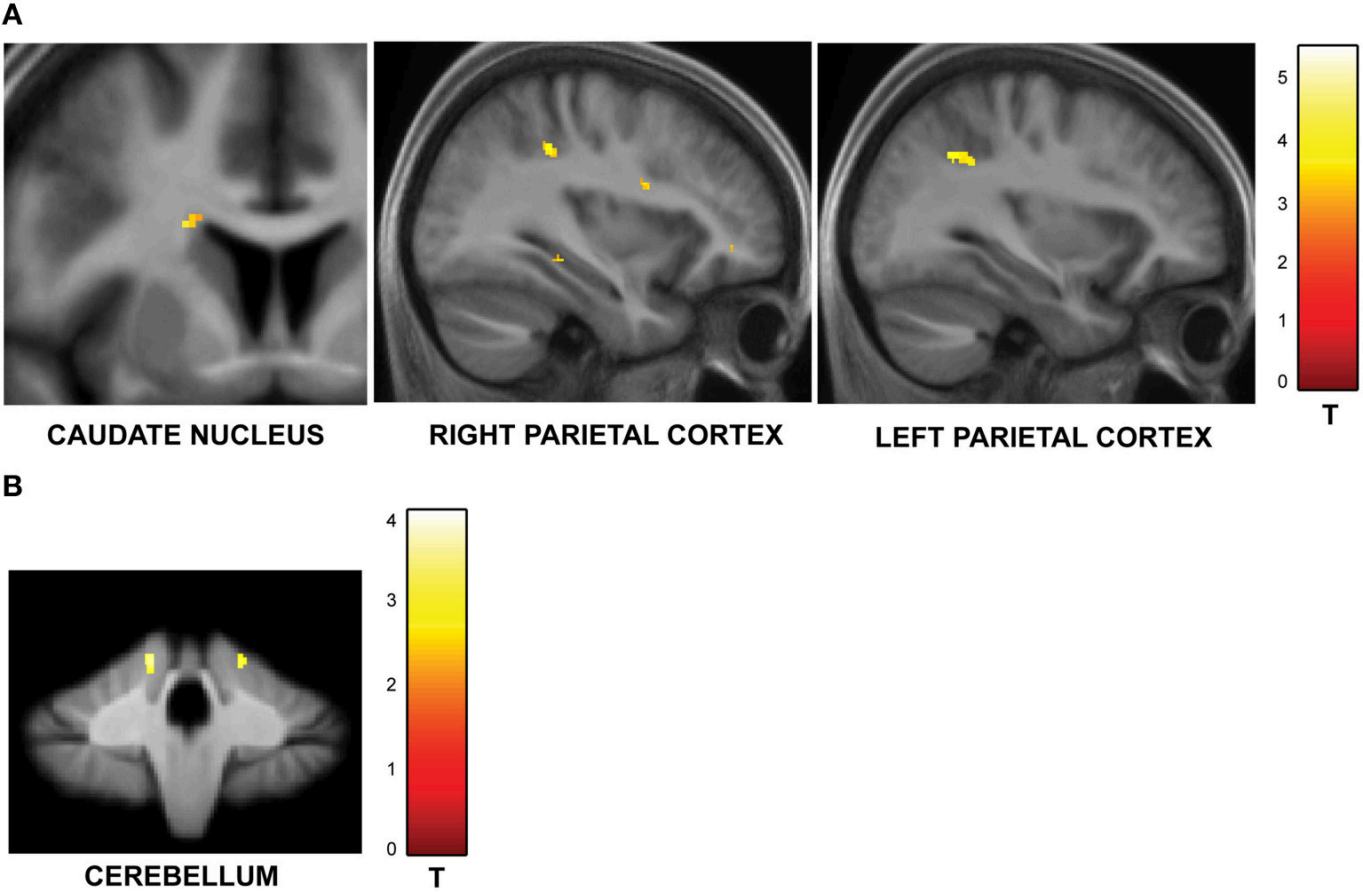
**Functional imaging results: effect of number of intervals. (A)** Brain areas that encode temporal memory as a function of increasing number of intervals. The activity in the caudate and the inferior parietal cortex was found to increase parametrically with the number of intervals. The MNI coordinates of these areas are listed in Table 2A. **(B)** Brain areas that encode temporal memory as a function of decreasing number of intervals. BOLD responses in the cerebellum was found to vary as a function of decreasing number of intervals. The MNI coordinates are provided in Table 2B.

The second question focused on parametric brain responses as a function of increasing numbers of intervals. Results across all subjects revealed significant activations in the bilateral inferior parietal cortex (abutting supramarginal gyrus) and the left caudate nucleus (Figure 3A; Table 2A). In the 12/16 subjects who showed a significant behavioral effect of number of intervals, similar activations in the inferior parietal cortex were observed as well (*x* = 33, *y* = −37, *z* = 39; *t* = 4.11, and *x* = −28, *y* = −52, *z* = 39, *t* = 3.97, respectively). As the number of intervals decreased, activity in the superior cerebellum increased as shown in Figure 3B. Other areas to encode memory for time with decreasing number of intervals included the inferior orbitofrontal cortex and the insula (also see Table 2B).

**Table 2A:** **Brain areas whose activity increased as a function of memory load**.

**Brain area**	**Hemisphere**	***x***	***y***	***z***	***t*-value**
Parietal cortex	Right	32	−36	38	4.88
	Left	−32	−54	36	3.70
Caudate nucleus	Left	−17	−18	20	3.92

*Local maxima are shown at p ≤ 0.001 (uncorrected)*.

**Table 2B:** **Brain areas whose activity decreased as a function of memory load**.

**Brain area**	**Hemisphere**	***x***	***y***	***z***	***t*-value**
Inferior Orbitofrontal cortex	Right	47	27	−6	4.03
Cerebellum lobule V	Right	26	−47	−20	3.82
Insula	Left	−35	6	11	3.52

*Local maxima are shown at p ≤ 0.001 (uncorrected)*.

#### Effect of task context

**Figure 4:**
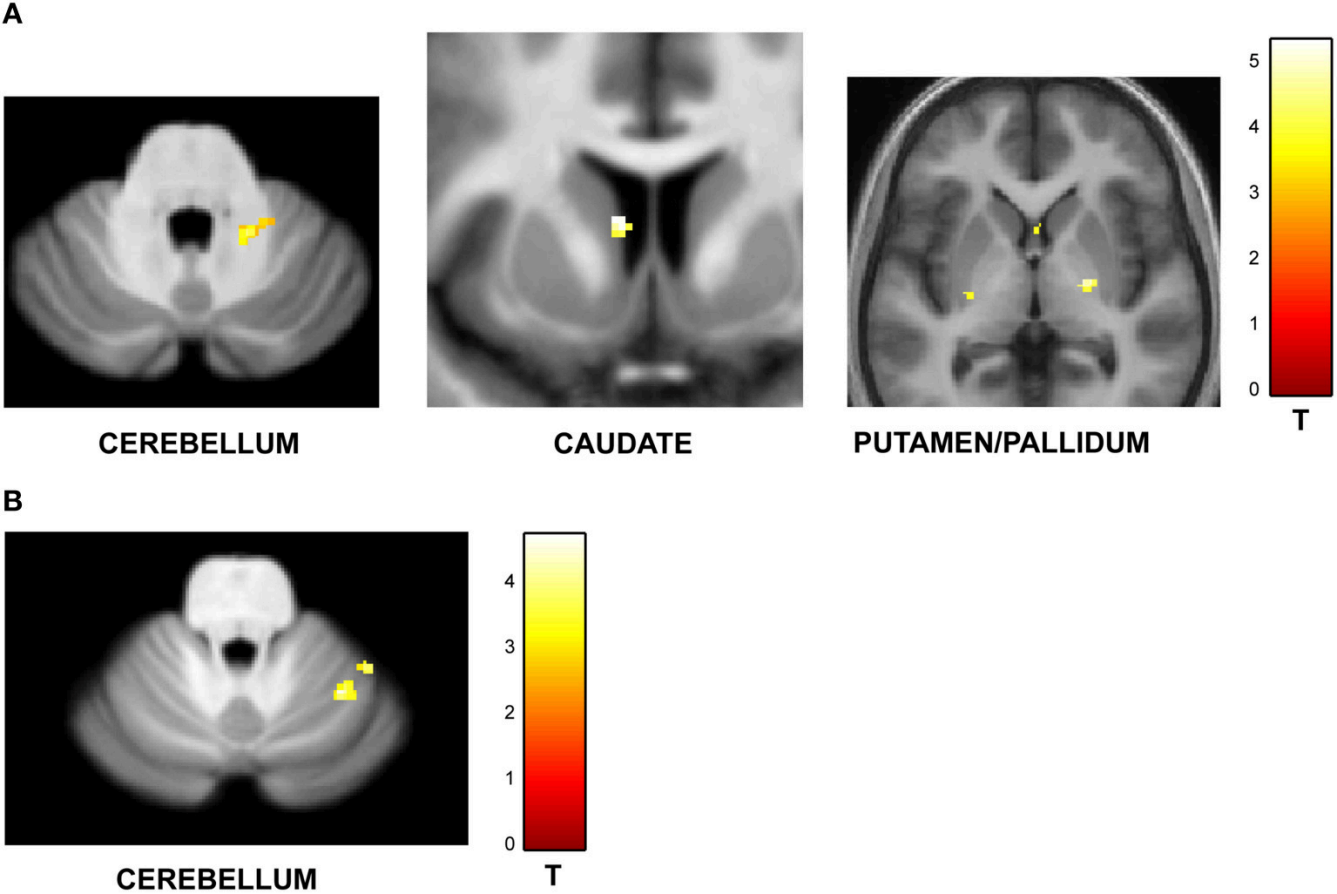
**Functional imaging results: effect of task context**. BOLD activations are shown for brain areas whose activity was found to be differentially modulated for trials with identical task structure (4 intervals with 20–25% jitter) but different context provided by the variable temporal structure in the jitter condition and the variable memory load in the number-of-intervals condition. All activations (except cerebellar activations on SUIT template) are displayed on the average normalized structural across all participants at a threshold of *p* < 0.001 (uncorrected). MNI coordinates and *t*-values are listed in Tables 3A,B respectively. **(A)** Brain areas with greater response for jitter vs. number of intervals condition. BOLD response in the cerebellum, caudate and putamen was found to be significantly modulated and higher during the jitter compared to the number-of-intervals condition for identical trials. **(B)** Brain areas with greater response for number of intervals vs. jitter condition. BOLD response in the cerebellum only was found to be higher for the identical trials in the number-of-intervals compared to the jitter condition.

One of the key motivations of the study was to examine whether encoding of time into memory depends on contextual factors like the temporal structure and number of intervals in the sequences. The experiment was designed to have an orthogonal design with 32 identical trials in the jitter and number-of-interval blocks respectively with a jitter of 20–25% and 4 intervals in each sequence. A subtraction analysis between jitter vs. number of interval blocks revealed enhanced activity in the right anterior cerebellar lobe and the striatum (including left caudate and bilateral putamen and pallidum) as shown in Figure 4A. Other areas included the thalamus, Heschl's gyrus, precuneus, hippocampus, orbitofrontal cortex, precuneus and the amygdala (see Table 3A). The reverse contrast (number of intervals vs. jitter) showed differential activation in the right cerebellar lobule VI (see Figure 4B; Table 3B).

**Table 3A:** **Brain areas activated for jitter vs. number-of-intervals condition**.

**Brain area**	**Hemisphere**	***x***	***y***	***z***	***t*–value**
Caudate	Right	15	11	11	4.43
	Left	−8	8	9	5.36
Pallidum	Right	24	−11	0	5.14
	Left	−21	11	4	4.07
Thalamus	Right	11	−26	14	4.93
	Left	−11	−26	9	4.52
Cerebellum Lobule V	Right	12	−54	−12	4.65
	Left	−8	−38	−8	4.26
Hippocampus	Right	23	−11	15	4.63
		−30	−14	−14	3.84
Putamen	Right	18	15	−3	3.81
	Left	−27	−17	1	4.24
Insula	Right	35	14	−3	4.11
	Left	−18	−1	12	3.67
Heschl's Gyrus	Left	−41	−29	9	4.05
Orbitofrontal cortex	Right	32	18	−21	3.76
	Left	−36	30	−6	4.03
Precuneus	Right	12	−41	42	3.99
Amygdala	Right	27	−1	−23	3.79
	Left	−26	−6	−18	3.86

*Local maxima are shown at p ≤ 0.001 (uncorrected)*.

**Table 3B:** **Brain areas activated for number-of-intervals vs. jitter condition**.

**Brain area**	**Hemisphere**	***x***	***y***	***z***	***t*-value**
Cerebellum Lobule VI	Right	32	−54	−24	4.59

*Local maxima are shown at p ≤ 0.001 (uncorrected)*.

### Structural imaging results

Structural imaging data were analyzed using VBM to investigate correlations between gray and white matter volume (GM; WM) and task performance. Specifically, we wanted to assess whether the key timing areas revealed by previous work (e.g., Grahn and Brett, 2007; Wiener et al., 2010b; Teki et al., 2011) and in the present study, i.e., the cerebellum and the striatum, also showed structural correlations with behavior. The correlations were performed between GM and WM density and precision (for all levels of the factor of interest, i.e., jitter and number of intervals).

**Figure 5:**
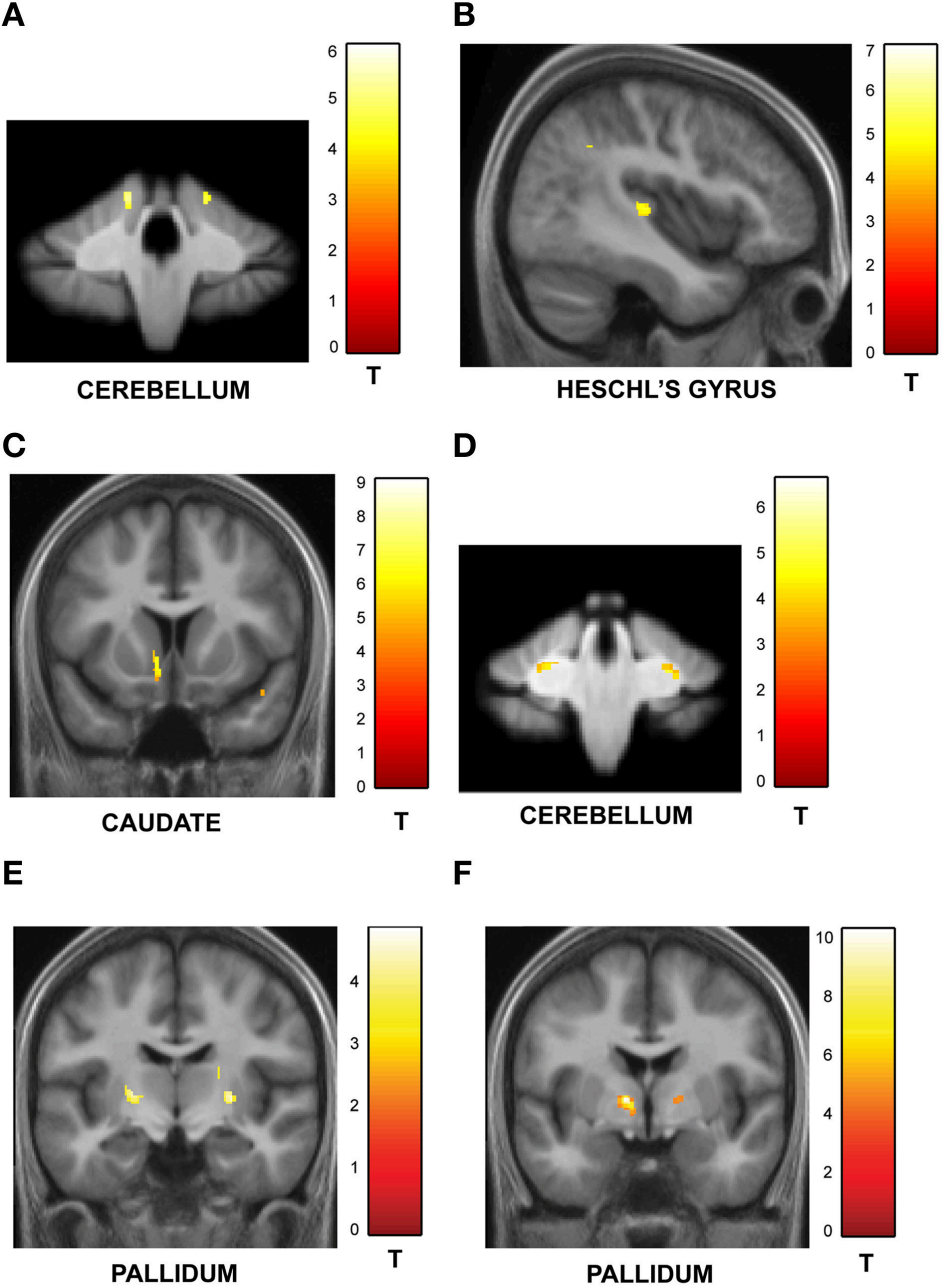
**Structural imaging results: correlation between GM and WM volume and behavior**. All activations are reported at a threshold of *p* < 0.001 (uncorrected) and scaled according to the *t*-value maps on the right. **(A)** Correlation between GM volume and performance on irregular sequences. The GM volume in the cerebellum increased with precision on irregular trials as shown here. Similar effects were observed in the orbitofrontal cortex and inferior temporal gyrus (Table S1A). **(B)** Correlation between GM volume and performance on regular sequences. The gray matter volume of sensory areas in Heschl's gyrus increased as a function of precision on regular sequences as shown here. Similar effects were also observed in the STG, insula and middle cingulate gyrus (Table S1B). **(C)** Correlation between GM volume and performance on sequences with high memory load. The gray matter volume of the caudate increased with performance on sequences with greater memory load. Correlations were also observed in the insula, thalamus and the Heschl's gyrus (as listed in Table S2A). **(D)** Correlation between GM volume and performance on sequences with low memory load. The cerebellum showed higher GM volume as a function of precision on sequences with low memory load (see Table S2B). **(E)** Correlation between WM volume and performance on irregular sequences. The pallidum expressed higher WM volume that correlated with listeners' precision as a function of increasing irregularity of the sequences (see Table S3A). **(F)** Correlation between WM volume and performance on sequences with high memory load. The pallidum showed higher WM volume that correlated with performance as a function of increasing memory load associated with the sequences (see Table S3B).

We found a significant correlation between precision on trials with increasing jitter and GM volume in the cerebellum (see Figure 5A) in a similar region of the cerebellar cortex as implicated in the functional data (Table S1A). In contrast, precision on trials with decreasing jitter and GM volume was demonstrated in sensory cortical areas including the Heschl's gyrus and superior temporal gyrus (see Figure 5B; Table S1B).

Similar analysis between precision on trials with increasing number of intervals and GM volume revealed significant clusters in the caudate (also activated in functional data) as shown in Figure 5C (also see Table S2A). The GM volume of the cerebellum was correlated with precision on trials with decreasing load (Figure 5D; Table S2B).

Correlation analysis of WM volume as a function of increasing jitter revealed bilateral clusters in the pallidum (Figure 5E; Table S3A) whilst no areas were found to be significant in the reverse contrast. The WM volume was also found to be higher in the pallidum as a function of increasing number of intervals (Figure 5F; Table S3B). The precuneus was the only area found to show significant effect in the opposite direction (Table S3C).

## Discussion

We investigated the neural bases of working memory for time intervals in the context of a shared resource model of working memory where the resource is flexibly distributed according to the amount of information to be encoded. We manipulated the information content in sequences by manipulating the temporal regularity and number of intervals, which we hypothesized to affect the working memory load. We examined, from first principles, whether there are core brain areas that are activated through these two manipulations of the resource even though the magnitude of the effect of temporal regularity and number of intervals may be different.

Behaviorally, listeners' performance decreased with greater information in the sequence, achieved by manipulating temporal jitter and the number of intervals. The fMRI activations revealed the striatum and cerebellum as core areas for encoding temporal memory as a function of increasing jitter and number of intervals. Additionally, the inferior parietal cortex was also strongly involved in representing time intervals as a function of load. We also analyzed structural correlations between gray and white matter volume and behavior that revealed correlations in the striatum and cerebellum, in line with the functional results. Furthermore, the analysis of context-specific responses for identical trials across the two conditions also revealed activations in the striatum and the cerebellum, suggesting on the whole, a critical role for these two subcortical motor areas in representing time intervals in working memory.

### Effect of jitter

Behavioral performance showed significant sensitivity to the temporal structure of the sequences (Figure 1B). The analyses of the underlying brain responses revealed activation of core timing areas in the cerebellum and the striatum (Buhusi and Meck, 2005; Ivry and Schlerf, 2008; Teki et al., 2011). Temporal context of the sequences of intervals provides a basis to distinguish the timing functions of the cerebellum and the striatum: whilst the cerebellum is associated with *absolute, duration-based* timing of intervals in irregular sequences, the striatum in coordination with fronto-striatal loops mediates *relative, beat-based* timing (Teki et al., 2012; Allman et al., 2014). This dissociation is supported by several lines of evidence: behavioral work (Monahan and Hirsh, 1990; Yee et al., 1994; Pashler, 2001; McAuley and Jones, 2003), neuropsychological assessment of patients (Grube et al., 2010; Cope et al., 2014a,b), motor timing studies (Schlerf et al., 2007; Spencer et al., 2007), and neuroimaging studies (Grahn and Brett, 2007; Teki et al., 2011; Grahn and Rowe, 2013). We have previously suggested a synergistic relationship between the striatum and the cerebellum whereby the striatum serves as a default clock and the cerebellum serves to encode the error in the timing activity of the striatal clock (Teki et al., 2012; Allman et al., 2014). Other timing models like the Striatal Beat Frequency model (SBF; Matell and Meck, 2004; Buhusi and Meck, 2005; Meck et al., 2008) based on coincident activity in the medium spiny neurons in the striatum do not address timing in sequences containing several intervals and the effect of temporal jitter.

The present data suggest that in addition to perception of time, the cerebellum and striatum also represent memory for time with the level of activation depending on the temporal context of the sequences. The cerebellum and vermis (see Table 1A for precise locations with cerebellum) were more strongly activated as a function of increasing jitter compared to the putamen and pallidum whilst the caudate and putamen were more active relative to the cerebellum as a function of decreasing jitter. Other memory-related areas that were activated as a function of increasing jitter included the precuneus, the posteromedial portion of the parietal lobe and the parahippocampal cortex. These two areas are involved in encoding and retrieval of episodic memory but have not been specifically implicated in temporal processing before. The activation of these areas suggests a link between subcortical timing areas and higher-order memory related areas in the medial temporal lobe that remains to be investigated.

It is important to note that sound-evoked activity is also observed in the cerebellum (e.g., Wolfe, 1972; Jastreboff and Tarnecki, 1975) and the basal ganglia (Hikosaka et al., 1989). Although it can be argued that the observed BOLD activations might capture sound-evoked responses, it is unlikely that such responses would scale as a function of jitter or number of intervals. Thus, the parametric analysis reported in the present study can be assumed to primarily reflect temporal processing activity.

### Effect of number of intervals

We also varied the amount of information in the sequences by manipulating the number of intervals. Although the task was based on the recall and reproduction of a single interval, the number-of-intervals condition required representation of multiple intervals in working memory. Activity in the caudate nucleus and the inferior parietal cortex systematically increased with increasing number of intervals in the sequence, consistent with previous event-related fMRI studies on memory for a single time interval (Rao et al., 2001; Coull et al., 2008).

The striatum is widely acknowledged to contribute to working memory (Postle and D'Esposito, 1999; Lewis et al., 2004; McNab and Klingberg, 2008; Darki and Klingberg, 2015) via dopaminergic interactions with frontal cortex (Goldman-Rakic, 1996; Frank et al., 2001). Consistent with this, disorders affecting the basal ganglia including Parkinson's, Huntington's and Multiple Systems Atrophy are associated with impairment on a range of working memory tasks (Robbins et al., 1992; Grahn et al., 2006; Dumas et al., 2013). The role of the striatum and frontal cortex in controlling access to working memory storage (McNab and Klingberg, 2008) is particularly significant in light of the SBF model that emphasizes the role of fronto-striatal dopaminergic loops in interval timing. The SBF model posits that striatal medium spiny neurons perform coincidence detection of cortical oscillatory activity, triggered by nigrostriatal dopaminergic signals. These theoretical considerations suggest a close relationship between perception and memory for time in fronto-striatal pathways (Darki and Klingberg, 2015).

The parietal cortex is also implicated in storage of information in working memory (McNab and Klingberg, 2008; Darki and Klingberg, 2015) and shows robust load-sensitive activity in visual working memory tasks (Todd and Marois, 2004; Vogel and Machizawa, 2004; Vogel et al., 2005; Ma et al., 2014). The parametric increase in the activity of the parietal cortex suggests a common framework for working memory processing in the brain that not only applies to storage of sensory information but also to temporal information. Timing activity in the parietal cortex has been demonstrated in nonhuman primates (Leon and Shadlen, 2003; Schneider and Ghose, 2012) as well as humans (Wiener et al., 2010a, 2012; Hayashi et al., 2015). Furthermore, the parietal cortex has also been shown to encode magnitude in general, and process time, space, and number (Walsh, 2003; Bueti and Walsh, 2009). The current data provide converging evidence from the temporal domain that parietal cortex may encode “temporal” magnitude and represent multiple time intervals in working memory.

The activity of the cerebellum (lobule V) was modulated as a function of decreasing load. This is consistent with cerebellar specialization for encoding the absolute duration of single intervals (Grube et al., 2010).

### Effect of task context

Behaviorally, there was no difference in precision between the trials that were identical in the jitter and number-of-intervals blocks (32 trials with 25% jitter and 4 intervals): *p* = 0.64, *t* = 0.47. However, there was a significant difference in BOLD responses between the two conditions. For a contrast of jitter vs. number-of-intervals, putamen, caudate, and cerebellum (lobule V) showed significant differential activity. The reverse contrast showed enhanced responses in the cerebellum (lobule VI) only. These data suggest that brain areas involved in holding and manipulating time intervals in memory are selectively activated by different task contexts: differential striatal and cerebellar activity for the jitter condition is consistent with previous work on rhythm and time perception (Grahn, 2012; Teki et al., 2012). The activation of cerebellar lobule VI is consistent with the specific role of this cerebellar sub-region in verbal working memory (Koziol et al., 2014), which may be attributed to its role in temporal sequencing of internal motor traces representing inner speech (Marvel and Desmond, 2010).

### Structural correlation with behavior

VBM correlation analysis was performed to assess whether the gray and white matter volume of specific temporal processing regions correlated with behavioral performance in the jitter and number-of-intervals conditions. In the absence of previous work on correlates between brain structure and timing behavior, we did not have strong well-defined anatomical hypotheses and, therefore, examined correspondence between the functional and structural brain data.

GM volume in the cerebellum (lobule V) correlated with behavior as the jitter increased, consistent with greater functional response in the same cerebellar sub-region. On the other hand, the GM volume of the Heschl's gyrus correlated with listeners' performance on regular trials. As the sequences become more regular, stronger phase-locking to the clicks at low rates (2 Hz) may explain the correlation observed in the auditory cortex. For the memory task, the GM volume of the caudate correlated with behavioral performance as the load increased. The reverse correlation was found in the cerebellum as a function of decreasing load.

Correlation between WM volume and behavior showed effects in the pallidum as a function of both increasing jitter and load. This result is consistent with recent evidence from a longitudinal study that revealed a correlation between working memory capacity and the fractional isotropy (FA) and the WM volume of fronto-striatal tracts (Darki and Klingberg, 2015). More specifically, they found that FA in white matter tracts and activity in the caudate predict future working memory capacity. Overall, the VBM results show strong correspondence with the functional data and highlight the importance of the cerebellum and the striatum in representation of temporal memory.

## Conclusions

We have demonstrated using fMRI that working memory for time intervals is implemented in a core resource in the striatum and the cerebellum, achieved through manipulating the information content by varying the regularity and number of intervals in sequences. These results are supported by concordant structural correlations with behavior in the same areas. Our results highlight functional and structural correlates of a flexible working memory resource for time intervals in rhythmic sequences and provide a strong basis to examine the underlying neural correlates of context-dependent memory for time, e.g., beta-band oscillations in the auditory-motor pathways (Iversen et al., 2009; Fujioka et al., 2012; Teki, 2014; Bartolo and Merchant, 2015), using techniques with higher temporal resolution than fMRI.

## Author contributions

ST designed the study; ST collected and analyzed the data; ST and TG wrote the manuscript.

### Conflict of interest statement

The authors declare that the research was conducted in the absence of any commercial or financial relationships that could be construed as a potential conflict of interest.
