# Supplementary material for: Brain Bases of Working Memory for Time Intervals in Rhythmic Sequences
Source: Front Neurosci. 2016 Jun 1;10:239. doi: 10.3389/fnins.2016.00239 (PMC4888525; doi:10.3389/fnins.2016.00239)
Supplement: Supplementary file 1 [file Table1.DOCX]

**Supplementary Tables**

**Table S1A. Brain areas which showed significant correlation between GM volume and performance on irregular sequences.**

| **Brain area** | **Hemisphere** | **x** | **y** | **z** | **t-value** |
| --- | --- | --- | --- | --- | --- |
| Inferior Temporal Gyrus | Right  Left | 51  -54 | -29  -57 | -27  -8 | 4.61  6.01 |
| Orbitofrontal Cortex | Right  Left | 17  −17 | 21  20 | −21  −24 | 5.60  5.49 |
| Cerebellum  Lobule V | Right  Left | 18  -11 | -38  -42 | -17  -17 | 5.13  4.89 |
| Precuneus | Right | 11 | -66 | 29 | 4.85 |
| Parahippocampal Gyrus | Right | 17 | -6 | -23 | 4.54 |

Local maxima are shown at p =< 0.0005 (uncorrected).

**Table S1B. Brain areas which showed significant correlation between GM volume and performance on regular sequences.**

| **Brain area** | **Hemisphere** | **x** | **y** | **z** | **t-value** |
| --- | --- | --- | --- | --- | --- |
| Middle Cingulate Gyrus | Right | 9 | -33 | 33 | 6.98 |
| Superior Temporal Gyrus | Right  Left | 56  −45 | -29  -21 | 7  0 | 6.30  6.16 |
| Insula | Right | 47 | -1 | -5 | 5.77 |
| Heschl’s Gyrus | Right  Left | 42  -36 | -24  -25 | 6  9 | 4.99  4.93 |

Local maxima are shown at p =< 0.0001 (uncorrected).

**Table S2A. Brain areas which showed significant correlation between GM volume and performance on sequences with high memory load.**

| **Brain area** | **Hemisphere** | **x** | **y** | **z** | **t-value** |
| --- | --- | --- | --- | --- | --- |
| Inferior Temporal Gyrus | Right | 48 | -38 | -24 | 9.04 |
| Caudate nucleus | Left | -6 | 11 | -9 | 7.52 |
| Insula | Right  Left | 38  -35 | -1  -6 | 6  7 | 7.35  7.01 |
| Thalamus | Right  Left | 8  -8 | -24  -25 | 6  4 | 6.53  4.98 |
| Rolandic Operculum | Right  Left | 46  -47 | -22  -28 | 15  15 | 5.11  6.17 |
| Amygdala | Right  Left | 23  -23 | -1  -1 | -14  -14 | 4.97  5.49 |
| Orbitofrontal cortex | Left | -12 | 23 | -24 | 5.17 |
| Heschl’s Gyrus | Right | 47 | -23 | 15 | 5.11 |

Local maxima are shown at p =< 0.001 (uncorrected).

**Table S2B. Brain areas which showed significant correlation between GM volume and performance on sequences with low memory load.**

| **Brain area** | **Hemisphere** | **x** | **y** | **z** | **t-value** |
| --- | --- | --- | --- | --- | --- |
| Superior Temporal Gyrus | Left | -51 | -32 | 7 | 6.69 |
| Middle Temporal Gyrus | Right | 54 | -23 | -17 | 5.17 |
| Hippocampus | Left | -36 | -15 | -15 | 5.10 |
| Cerebellum lobule VI | Right  Left | 23  -21 | -39  -45 | -33  -32 | 4.97  4.78 |

Local maxima are shown at p =< 0.001 (uncorrected).

**Table S3A. Brain areas which showed significant correlation between WM volume and performance on irregular sequences.**

| **Brain area** | **Hemisphere** | **x** | **y** | **z** | **t-value** |
| --- | --- | --- | --- | --- | --- |
| Pallidum | Right  Left | 26  -21 | -11  -12 | 3  4 | 4.90  4.36 |

Local maxima are shown at p =< 0.0007 (uncorrected).

**Table S3B. Brain areas which showed significant correlation between WM volume and performance on regular sequences.**

| **Brain area** | **Hemisphere** | **x** | **y** | **z** | **t-value** |
| --- | --- | --- | --- | --- | --- |
| Pallidum | Right  Left | -11  15 | -3  -1 | 1  1 | 10.36  5.12 |
| Thalamus | Right  Left | 15  −12 | -15  -14 | 12  0 | 8.85  6.12 |
| Middle Temporal Gyrus | Right | 47 | -51 | 6 | 9.53 |

Local maxima are shown at p =< 0.0005 (uncorrected).

**Table S3C. Brain areas which showed significant correlation between WM volume and performance on sequences with low memory load.**

| **Brain area** | **Hemisphere** | **x** | **y** | **z** | **t-value** |
| --- | --- | --- | --- | --- | --- |
| Precuneus | Right | 20 | -47 | 8 | 4.68 |

Local maxima are shown at p =< 0.001 (uncorrected).
